# Computed tomography-measured body composition: correlation with postoperative morbidity and mortality in patients with gastroesophageal cancer

**DOI:** 10.1590/0100-3984.2019.0009

**Published:** 2019

**Authors:** Almir Galvão Vieira Bitencourt, Thais Manfrinato Miola, Juliana de Oliveira Souza, Elizabeth Launeir Santos da Conceição, Felipe José Fernandez Coimbra, Paula Nicole Vieira Pinto Barbosa

**Affiliations:** 1 A.C.Camargo Cancer Center, São Paulo, SP, Brazil.; 2 Universidade Nove de Julho, São Paulo, SP, Brazil.

**Keywords:** Gastrointestinal neoplasms, Stomach neoplasms, Esophageal neoplasms, Body composition, Body fat distribution, Tomography, X-ray computed

## Abstract

**Objective:**

To determine whether preoperative anthropometric and computed tomography (CT) measurements of body composition can predict postoperative morbidity and mortality in patients with gastric or esophageal cancer.

**Materials and Methods:**

This was a retrospective study in which we reviewed the medical records and abdominal CT scans of patients with gastric or esophageal cancer who underwent surgery in 2015 at a cancer center. CT scans performed during routine preoperative evaluation were retrospectively assessed to measure the area of lean body mass at the level of the third lumbar vertebra, as well as the area of visceral and subcutaneous fat.

**Results:**

Seventy patients were included in the study. The mean age was 59.9 years (range, 33-82 years), and 47 patients (67.1%) were men. The mean postoperative follow-up period was 14.9 months. Neither postoperative morbidity nor postoperative mortality correlated significantly with gender, age, the type of primary tumor, the presence of comorbidities, smoking status, body mass index, nutritional status, or visceral fat area. The survival rate was higher for patients with normal lean body mass than for those with low lean body mass (hazard ratio = 0.116; 95% confidence interval: 0.015-0.906; *p* = 0.040).

**Conclusion:**

Our data suggest that lean body mass can be a relevant prognostic factor in patients with gastric or esophageal cancer, and that CT measurements should be included in the routine preoperative evaluation, because it may provide information that aids nutritional and clinical care for these patients.

## INTRODUCTION

The assessment of nutritional status should be considered an integral part of cancer treatment. Malnourished patients with cancer show a poorer response to therapeutic interventions, a higher incidence of complications, longer hospital stays, poorer immunological status, poorer quality of life, higher morbidity, and higher mortality than do well-nourished patients^(1)^. Malnutrition, which is quite common among patients with gastric or esophageal cancer, has a multifactorial etiology, which may be related to dysphagia, cachexia, or therapeutic interventions^(2)^.

Weight loss in patients with cancer is mainly associated with sarcopenia, which is defined as loss of muscle mass, with or without loss of adipose tissue, and decreased functional ability. According to European consensus criteria, sarcopenia can be classified into to three stages as follows: presarcopenia, when only lean body mass is reduced; sarcopenia, when reduced lean body mass is accompanied by either reduced muscle strength or reduced physical performance; and severe sarcopenia, when reduced lean body mass is accompanied by reduced muscle strength and reduced physical performance^(3)^.

The use of imaging methods to assess body composition has been increasing because they are more accurate than are conventional anthropometric measures and bioelectrical impedance analysis^(4-6)^. Densitometry, computed tomography (CT), and magnetic resonance imaging are the most commonly used imaging techniques. In patients with cancer, CT has been prioritized, because it can be used for disease staging and for the assessment of treatment response, as well as for the measurement of lean body mass, visceral fat, and subcutaneous fat^(7,8)^.

This study aimed to determine whether preoperative anthropometric and CT measurements of body composition can predict postoperative morbidity and mortality in patients with gastric or esophageal cancer.

## MATERIALS AND METHODS

In this retrospective study, we reviewed the medical records and abdominal CT scans of 99 patients with gastric or esophageal cancer who underwent surgery in 2015 at a referral center for cancer. The study was approved by the research ethics committee of our institution prior to data collection. We included patients who had undergone abdominal CT within the last three months prior to surgery and for whom nutritional data were available.

We collected anthropometric data, including weight, height, body mass index (BMI), mid-arm muscle circumference, and triceps skinfold thickness. A tape measure and a Lange skinfold caliper (Cambridge Scientific Industries, Cambridge, MD, USA) were used in order to assess mid-arm muscle circumference and triceps skinfold thickness. Patients ≤ 60 years of age were classified according to standards proposed by Frisancho^(9)^, whereas those > 60 years of age were classified according to reference ranges proposed by Kuczmarski et al.^(10)^. We calculated the BMI by dividing the weight (in kilograms) by the height (in meters) squared (kg/m^2^). The World Health Organization reference ranges were used in order to classify the BMIs of patients ≤ 60 years of age, whereas the Pan American Health Organization reference ranges were used for those of patients > 60 years of age^(11)^. The nutritional status was based on a compilation of these measures.

**Figure 1:**
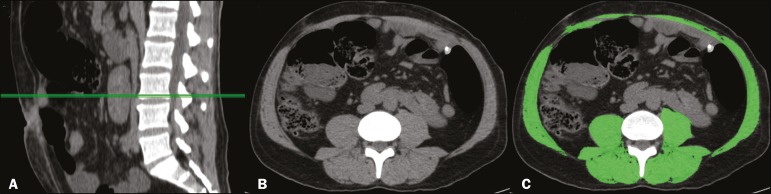
CT measurements of lean body mass. **A:** Sagittal plane used in order to set the axial plane at the level of the inferior endplate of the third lumbar vertebral body (**B**). **C:** Segmentation of the lean body mass at the same level as **B**.

**Figure 2:**
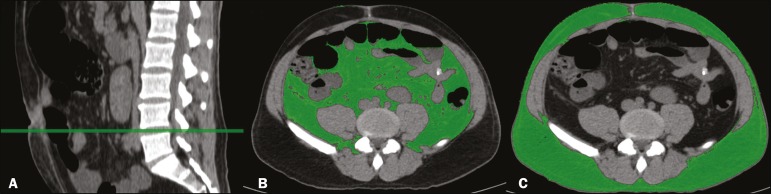
CT measurements of visceral and subcutaneous fat. **A:** Sagittal plane used in order to set the axial plane between the fourth and fifth lumbar vertebral bodies. **B,C**: Segmentation of visceral and subcutaneous fat (**B** and **C**, respectively) at the same level.

In accordance with the abdominal surgery protocol of our institution, CT scans had been performed during the routine preoperative evaluation, and we evaluated those scans retrospectively. All CT examinations were performed in a 16-multidetector scanner (Brilliance Big Bore; Philips Healthcare, Cleveland, OH, USA), and all CT images were reviewed by the same radiologist. To determine body composition, on the basis of previously validated, widely used parameters^(8)^, we employed image processing software (OsiriX; Pixmeo, Geneva, Switzerland). Unenhanced axial CT scans of the abdomen were used in order to measure the surface area of lean body mass (skeletal muscles, including the psoas muscle, paravertebral muscles, and abdominal wall muscles) at the level of the inferior endplate of the third lumbar vertebra, and the area of visceral and subcutaneous fat at the level of the fourth and fifth lumbar vertebrae (Figures 1 and 2). If required, a semi-automated method with manual correction was used in order to identify adipose tissue (defined as tissue with a density from −190 HU to −30 HU) and skeletal muscles (defined as tissue with a density from −29 HU to +150 HU), using the Segmentation Plugin for the OsiriX 2D/3D Viewer (Computer Aided Medical Procedures, Technische Universität München and Department of Nuclear Medicine, University Hospital Rechts der Isar, Munich, Germany). The visceral fat area was divided into the following categories, as proposed by Murray et al.^(8)^: < 100 cm^2^; 100-130 cm^2^; and > 130 cm^2^. Lean body mass area was corrected for height (lean body mass in cm^2^/height in m^2^) to calculate the lean BMI (LBMI). Lean body mass was considered low when the LBMI at the level of the third lumbar vertebra was < 55.4 cm^2^/m^2^ for men or < 38.9 cm^2^/m^2^ for women^(12)^.

To assess postoperative morbidity and mortality, we evaluated the following variables: length of intensive care unit (ICU) stay in the immediate postoperative period; overall length of hospital stay; complications requiring reoperation or readmission to the ICU; date of last follow-up visit; in-hospital mortality; and mortality after discharge.

For data analysis, we used the SPSS Statistics software package, version 20.0 (IBM Corp.; Armonk, NY, USA). Descriptive statistics were calculated as measures of central tendency (mean, median, and mode) and dispersion (standard deviation and range) or as absolute and relative frequencies. Statistical tests were used, when required, to identify correlations between variables. The chi-square test and Fisher’s exact test were used in the comparison of categorical variables; the Student’s t-test was used for continuous variables with normal distribution; and the Mann-Whitney U test was used for continuous variables without normal distribution. The Kaplan-Meier curve was used for survival analysis. The log-rank test was used for the comparison of survival curves among different groups, whereas Cox regression was used in order to estimate hazard ratios with the respective 95% confidence intervals (95% CIs). The level of significance was set at 5%.

## RESULTS

We reviewed the records of 99 patients with gastric or esophageal cancer. Of those, 29 were excluded, either because the nutritional data were not available or because the patient had not undergone abdominal CT within the last three months prior to surgery. Therefore, the final sample comprised 70 patients: 52 (74.3%) with gastric cancer; and 18 (25.7%) with esophageal cancer. Fifty-four patients (77.1%) underwent neoadjuvant chemotherapy. The mean age was 59.9 ± 11.8 years (range, 33-82 years), and 47 patients (67.1%) were men. The most common comorbidities were hypertension, in 21 (30.0%); diabetes, in 11 (15.7%); and dyslipidemia, in 3 (4.3%). Thirty-five patients (50.0%) had never smoked, 21 (30.0%) were former smokers, and 14 (20.0%) were current smokers.

A descriptive analysis of the quantitative variables assessed in the anthropometric measurement of nutritional status and CT measurement of body composition is shown in Table 1. According to the nutritional assessment based on BMI, 39 (55.7%) of the 70 patients were of normal weight, whereas 14 (20.0%) were overweight, 9 (12.9%) were obese, and 8 (11.4%) were underweight. On the basis of mid-arm muscle circumference, we categorized 31 (44.3%) of the patients as malnourished and 35 (50.0%) as well-nourished. According to the nutritional diagnosis, 26 (37.1%) of the patients were malnourished, 25 (35.7%) were well-nourished, 11 (15.7%) were overweight, and 8 (11.4%) were obese. According to the CT-measured LBMI, 38 (54.3%) of the patients had low lean body mass. The visceral fat area was < 100 cm^2^ in 29 patients (42.0%), 100-130 cm^2^ in 10 (14.5%), and > 130 cm^2^ in 30 (43.5%).

**Table 1:** Descriptive analysis of quantitative variables assessed in an anthropometric measurement of nutritional status and CT measurement of body composition in patients with gastric or esophageal cancer (n = 70).

Variable	Mean	Range	SD
Nutritional assessment			
Weight (kg)	70.0	45.0-117.6	15.3
Height (m)	1.7	1.40-1.87	0.1
BMI (kg/m^2^)	25.3	24.2-38.8	4.7
Triceps skinfold thickness (mm)	16.5	5.0-43.0	8.6
Triceps skinfold thickness (%)	98.8	6.0-226.0	45.0
Mid-arm muscle circumference (cm)	23.9	18.10-29.70	3.1
Mid-arm muscle circumference (%)	90.1	2.0-129.0	16.3
CT-measured body composition			
Lean body mass (cm^2^)	133.6	80.4-238.1	31.8
LBMI (cm^2^/m^2^)	47.8	29.2-78.6	8.6
Visceral fat area (cm^2^)	123.8	13.7-347.2	67.8
Subcutaneous fat area (cm^2^)	212.8	25.4-591.0	112.7

SD, standard deviation.

Of the 70 patients evaluated, 63 (90.0%) required ICU admission in the immediate postoperative period. The mean postoperative ICU stay was 3.30 ± 3.0 days (range, 1-19 days), and the mean overall hospital stay was 13.22 ± 11.9 days (range, 1-65 days). Postoperative complications occurred in 11 patients (15.7%), all of whom required reoperation or ICU readmission. Four patients (5.7%) died during their hospital stay. We found that length of ICU stay, postoperative complication rates, and in-hospital mortality were not significantly associated with gender, age, the presence of comorbidities, smoking status, the type of primary tumor, BMI, nutritional status, LBMI, or the visceral fat area.

**Figure 3:**
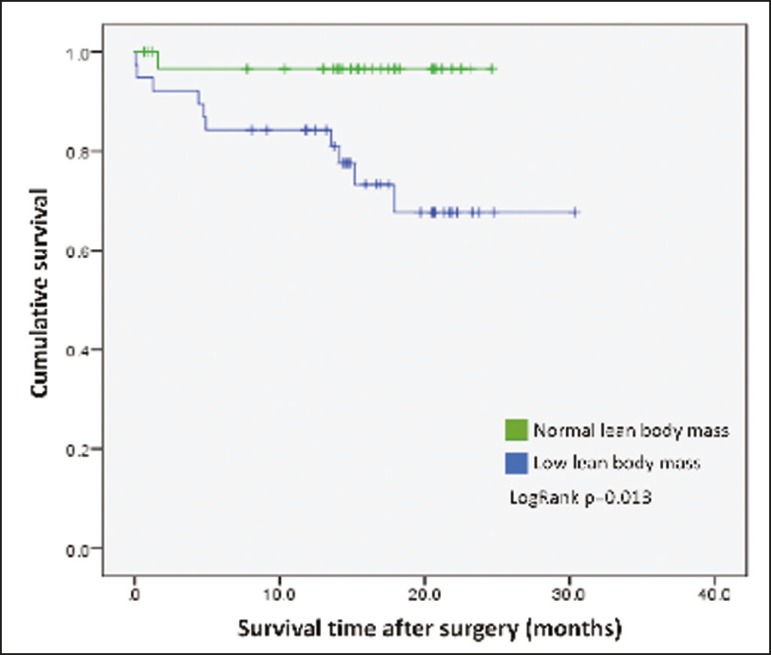
Survival curve according to a classification of CT-measured LBMI in patients with gastric or esophageal cancer.

The mean postoperative follow-up was 14.9 ± 6.9 months (range, 0.1-30.3 months), and 11 patients (15.7%) died during the study period (4 died during their hospital stay and 7 died after discharge). The survival rate was higher for patients with normal lean body mass, based on CT-measured LBMI, than for those with low lean body mass (hazard ratio = 0.116; 95% CI: 0.015-0.906; *p* = 0.040) (Figure 3). Of the 11 patients who died during the study, 10 had low lean body mass and only 1 had normal lean body mass, the difference being statistically significant (*p* = 0.009). Survival was not significantly associated with gender, age, type of primary tumor, comorbidities, histological type of the tumor, neoadjuvant chemotherapy, smoking status, BMI, nutritional diagnosis, or visceral fat area.

## DISCUSSION

Our results indicate that CT-measured lean body mass is an important prognostic factor in patients with gastric or esophageal cancer. More than half of our patients had low lean body mass at the preoperative evaluation. Loss of lean body mass may be caused by the disease itself or by neoadjuvant chemotherapy, which was performed in approximately 77% of our sample. Although the complication rates, length of hospital stay, and in-hospital mortality rates did not differ significantly between the patients with low lean body mass than among those with normal lean body mass, overall survival was lower among the former. Anthropometric characteristics and visceral fat measures did not correlate with postoperative morbidity and mortality.

As in our study, Tegels et al.^(13)^ assessed patients with gastric cancer (n = 152) and found a high (57.7%) prevalence of low lean body mass, which, however, was not associated with postoperative complications, in-hospital mortality, or 6-month mortality. Other authors have found increased postoperative complication rates and poorer prognoses in patients with gastric cancer and low lean body mass^(14-17)^. Kuwada et al.^(18)^ showed that, in patients with gastric cancer, sarcopenia associated with comorbidities increases the risk of death due to causes other than cancer.

In a study of 252 patients with locally advanced esophageal cancer, Elliott et al.^(19)^ found that CT-measured lean body mass correlated with increased postoperative complications, although not with survival. The authors also showed that the proportion of patients with low lean body mass increased from 16% to 31% after neoadjuvant chemotherapy, with no significant change in body fat. Loss of lean body mass during neoadjuvant chemotherapy is also associated with higher morbidity and mortality in patients with esophageal cancer, especially at the more advanced stages^(20,21)^. Paireder et al.^(22)^ assessed 130 patients with advanced esophageal cancer who underwent neoadjuvant chemotherapy and found that low lean body mass at the end of treatment was associated with poorer long-term survival. Low lean body mass at diagnosis is also related to a higher risk of neoadjuvant chemotherapy toxicity in patients with gastric or esophageal cancer^(23-25)^.

Most patients with gastric or esophageal cancer undergo CT scanning for disease staging at diagnosis. Lean body mass can be evaluated by using CT scans, without the need for additional procedures or radiation. Some authors have suggested that lean body mass measurement be part of the routine preoperative evaluation of patients with gastrointestinal tract cancer because of its prognostic value^(18)^. In addition, preliminary studies indicate that preoperative nutritional support and physical activity increase lean body mass, as well as reducing postoperative morbidity and mortality^(26)^.

Our study has some limitations. First, the retrospective nature of the study led to the exclusion of some patients due to a lack of nutritional data or CT scans for assessment. In addition, it was not possible to recover important information systematically for this study, such as clinical staging, which could have been included as a confounder in the statistical analysis. Second, the relatively small sample may have hindered some statistical analyses. Furthermore, we did not assess functional changes related to low lean body mass, which are important for the diagnosis and classification of sarcopenia in this population. In a study of 470 patients with gastric cancer, Huang et al. showed prevalence rates of 20.6%, 10%, and 6.8% for presarcopenia, sarcopenia and severe sarcopenia, respectively, as well as increased postoperative complication rates in all three of those groups^(27)^.

In conclusion, low preoperative CT-measured lean body mass can be used as a relevant prognostic factor in patients with gastric or esophageal cancer. In our sample, survival was poorer among the patients with low CT-measured lean body mass than among those with normal lean body mass. Our data suggest that CT measurements of lean body mass should be included in the routine preoperative assessment of patients with gastric or esophageal cancer, because they may provide information that informs the nutritional and clinical care of such patients.
